# Occurrence, Typing, and Resistance Genes of ESBL/AmpC-Producing *Enterobacterales* in Fresh Vegetables Purchased in Central Israel

**DOI:** 10.3390/antibiotics12101528

**Published:** 2023-10-11

**Authors:** Hadas Kon, Mor Lurie-Weinberger, Adi Cohen, Liat Metsamber, Alona Keren-Paz, David Schwartz, Yehuda Carmeli, Vered Schechner

**Affiliations:** 1 National Institute for Antibiotic Resistance and Infection Control, Ministry of Health, Tel-Aviv 6423906, Israel; hadaskon@tlvmc.gov.il (H.K.); morlw@tlvmc.gov.il (M.L.-W.); adicoh@tlvmc.gov.il (A.C.); alonakp@tlvmc.gov.il (A.K.-P.); davidsc@tlvmc.gov.il (D.S.); yehudac@tlvmc.gov.il (Y.C.); 2 School of Public Health, Faculty of Medicine, Tel-Aviv University, Tel-Aviv 6997801, Israel; liat.metsamber@gmail.com; 3 Faculty of Medicine, Tel-Aviv University, Tel-Aviv 6997801, Israel

**Keywords:** AmpC beta-lactamase, antibiotic resistance, *Enterobacter cloacae*, ESBL, *Escherichia coli*, food pathogens, food safety, Fourier-transform infrared spectroscopy, Klebsiella pneumoniae, whole genome sequencing

## Abstract

Beta-lactam resistance can lead to increased mortality, higher healthcare expenses, and limited therapeutic options. The primary mechanism of beta-lactam resistance is the production of extended-spectrum beta-lactamases (ESBL) and AmpC beta-lactamases. The spread of beta-lactamase-producing *Enterobacterales* via the food chain may create a resistance reservoir. The aims of this study were to determine the prevalence of ESBL/AmpC-producing *Enterobacterales* in vegetables, to examine the association between EBSL/AmpC-producing bacteria and types of vegetables, packaging, and markets, and to investigate the genetic features of ESBL-producing isolates. The antibiotic susceptibilities were determined using VITEK. Phenotypic ESBL/AmpC production was confirmed using disk diffusion. ESBL-producing isolates were subjected to Fourier-transform infrared (FT-IR) spectroscopy and to whole genome sequencing using Oxford Nanopore sequencing technology. Of the 301 vegetable samples, 20 (6.6%) were positive for ESBL producers (16 *Klebsiella pneumoniae* and 4 *Escherichia coli*), and 63 (20.9%) were positive for AmpC producers (56 *Enterobacter cloacae* complex, 4 *Enterobacter aerogenes*/*cancerogenus*, and 3 *Pantoea* spp., *Aeromonas hydrophila*, and *Citrobacter braakii*). The *bla*CTX-M and *bla*SHV genes were most common among ESBL-producing isolates. The beta-lactamase genes of the ESBL producers were mainly carried on plasmids. Multilocus sequence typing and FT-IR typing revealed high diversity among the ESBL producers. AmpC producers were significantly more common in leafy greens and ESBL producers were significantly less common in climbing vegetables. The presence of ESBL/AmpC-producing *Enterobacterales* in raw vegetables may contribute to the dissemination of resistance genes in the community.

## 1. Introduction

Bacteria carrying gene coding for extended-spectrum beta-lactamases (ESBL) and AmpC beta-lactamases exhibit hydrolytic activity in response to numerous beta-lactam antibiotics, resulting in ineffective antibiotic treatment. Beta-lactam antibiotics are the mainstay of treatment against many infections caused by Gram-negative bacteria [1]. Therefore, the spread of ESBL/AmpC-producing Gram-negative bacteria poses a growing concern worldwide and is of clinical and epidemiological importance [2]. The case fatality rate for ESBL-producing *Enterobacterales* infections ranges between 12% and 41% [3], and the in-hospital costs of a single ESBL infection reach up to US$30,093 [4]. Similarly, higher mortality and increased hospital costs were observed for patients infected with AmpC-producing bacteria compared with susceptible isolates of the same organisms [5].

The spread of antimicrobial resistance occurs through the dissemination of clones or via horizontal gene transfer. The most prevalent and clinically relevant ESBL enzymes are TEM (Temoneira, named after the patient infected with the first isolate expressing TEM-1), SHV (sulfhydryl reagent variable), and CTX-M (cefotaxime-hydrolyzing beta-lactamase isolated in Munich), with CTX-M enzymes emerging as the most common [6]. While TEM- and SHV-type ESBLs are highly related, differing by only a few amino acid substitutions, CTX-M-type ESBLs are more genetically diverse [6]. Most ESBL-encoding genes are located on plasmids. The resistance-conferring plasmids of incompatibility (Inc) groups IncF, IncI1, and IncC are associated with ESBL-producing *Enterobacterales*. IncF plasmids are the predominant group, and can be further categorized into IncFII, IncFIA, and IncFIB [7,8]. AmpC beta-lactamases are clinically important cephalosporinase class C enzymes, which are commonly chromosomally encoded in certain species, but may also be plasmid-mediated [9].

ESBL-producing *Enterobacterales* were originally considered nosocomial pathogens and the main reservoir of carriers was among patients hospitalized in acute care and post-acute care facilities. In the last two decades, infections with these resistant organisms have been increasingly reported in outpatient settings [10]. The appearance of resistant *Enterobacterales* in the community could be the result of spread from healthcare institutions into the community, or could stem from an environmental source (e.g., domestic animals or contaminated food) [10]. Raw vegetables may constitute a source of AmpC/ESBL-producing *Enterobacterales*. Possible contamination pathways include contaminated biological fertilizer or water, poor hygiene practices of the workers involved in food production, ineffective food safety management, or unsanitary vegetable packaging or display [11,12].

In Israel, third-generation cephalosporin resistance (3GCR) in community onset infections is common, and reaches almost 30% among *Escherichia coli* causing community-onset bacteremia [13]. There are no available data on the prevalence of ESBL/AmpC in vegetables in Israel. Worldwide, the prevalence of ESBL-positive isolates in fresh vegetables varies between 0.06 and 25% [14,15,16,17,18,19] and the range of AmpC-positive isolates is 0.5–14% [15,17,20,21].

In this study, we aimed to determine the prevalence of ESBL/AmpC-producing *Enterobacterales* in fresh vegetables in Israel and to examine the association between EBSL/AmpC-producing bacteria and types of vegetables, packaging, and markets. In addition, we aimed to analyze the genetic features of ESBL-producing isolates in order to investigate their resistance genes and plasmid carriage status.

## 2. Results

**Figure 1:**
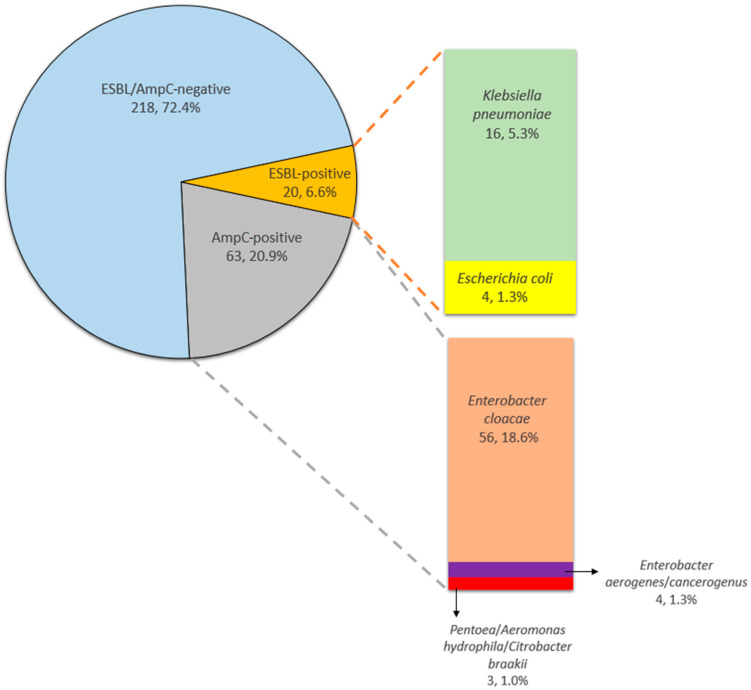
Prevalence of ESBL- and AmpC-producing *Enterobacterales* and distribution according to bacteria.

**Figure 2:**
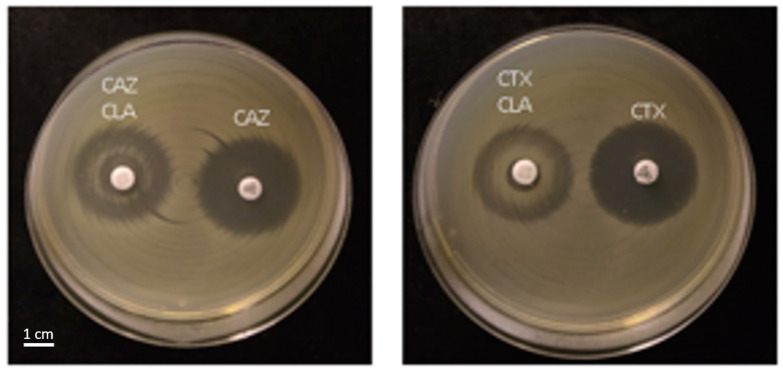
AmpC induction exhibited by disk diffusion with ceftazidime/ceftazidime-clavulanate (CAZ/CAZ-CLA; (**left**)) and cefotaxime/cefotaxime-clavulanate (CTX/CTX-CLA; (**right**)) in one representative *Enterobacter cloacae* strain.

Out of the 301 vegetables sampled, 20 (6.6%, 95% CI 4.1–10.1%) were positive for ESBL-producing *Enterobacterales*, including *Klebsiella pneumoniae* (*n* = 16) and *E. coli* (*n* = 4) (Figure 1). The prevalence of samples positive for ESBL-producing *E. coli* was 1.3% and that of ESBL-producing *K. pneumoniae* was 5.3%. 63 samples contained AmpC-producing isolates (20.9%, 95% CI 16.8–26.0%), and were identified as *Enterobacter cloacae* complex (*n* = 56), *Enterobacter aerogenes*/*cancerogenus* (*n* = 4), *Pantoea* spp. (*n* = 1), *Aeromonas hydrophila* (*n* = 1), and *Citrobacter braakii* (*n* = 1) (Figure 1). Of the 63 AmpC-positive isolates, 6 *Enterobacter* spp. isolates exhibited AmpC induction (Figure 2). A total of 218 Gram-negative bacteria were negative for ESBL and AmpC, of which 63 samples showed no growth on the selective agar plate, and 155 grew on the selective agar but tested negative for ESBL/AmpC, including *Citrobacter* spp. (*n* = 22), carbapenem-susceptible *Acinetobacter* spp. (*n* = 58), *Pseudomonas* spp. (*n* = 72), *Enterobacter* spp. (*n* = 1), *Leclercia adecarboxylata* (*n* = 1), and *Raoultella ornithinolytica* (*n* = 1) (Figure 1).

The distribution of the sample according to vegetable types, packaging, and market types is presented in Table 1. AmpC-producing bacteria were most prevalent among leafy greens, while ESBL-producing *Enterobacterales* were least common in climbing vegetables.

All 20 ESBL-producing isolates were resistant to ampicillin, cefazolin, cefuroxime, and ceftriaxone, whereas resistance to ceftazidime varied. All were susceptible to cefoxitin and carbapenems (meropenem and ertapenem) and the majority were susceptible to aminoglycosides (amikacin and gentamicin). Only one isolate was susceptible to ciprofloxacin (Table S1).

Whole genome sequencing (WGS) revealed that all ESBL-producing isolates harbored at least one ESBL variant (*bla*TEM, *bla*SHV, and *bla*CTX-M) in different combinations. The *bla*CTX-M, *bla*SHV, and *bla*TEM genes were detected in 95%, 75%, and 50% of the ESBL isolates, respectively. The predominant allele of *bla*CTX-M was *bla*CTX-M-15. Six (30%) ESBL-positive *K. pneumoniae* isolates exhibited co-occurrence of all three families (*bla*TEM+ *bla*SHV+ *bla*CTX-M). Two ESBL isolates carried *bla*CTX-M without an additional *bla* gene, whereas co-occurrence of *bla*TEM+ *bla*SHV, *bla*CTX-M+ *bla*TEM, and CTX-M+ *bla*SHV was found in one, three, and eight ESBL-producing isolates, respectively (Table S2). All ESBL-producing isolates carried genes conferring resistance to aminoglycosides and fluoroquinolones, excluding one ESBL-producing *E. coli* isolate which did not carry any aminoglycoside-resistant genes. Three *K. pneumoniae* ESBL-producing isolates harbored the *aac(6′)-Ib-cr* gene, which confers resistance to both aminoglycosides and fluoroquinolones. All ESBL-producing *K. pneumoniae* isolates carried *fosA*, a resistance determinant to fosfomycin, whereas *fosA* was not identified in any *E. coli* ESBL-producing isolates. Genes conferring resistance to disinfectants and quaternary ammonium compounds were identified in 50% of the ESBL-producing isolates (Table S2).

**Figure 3:**
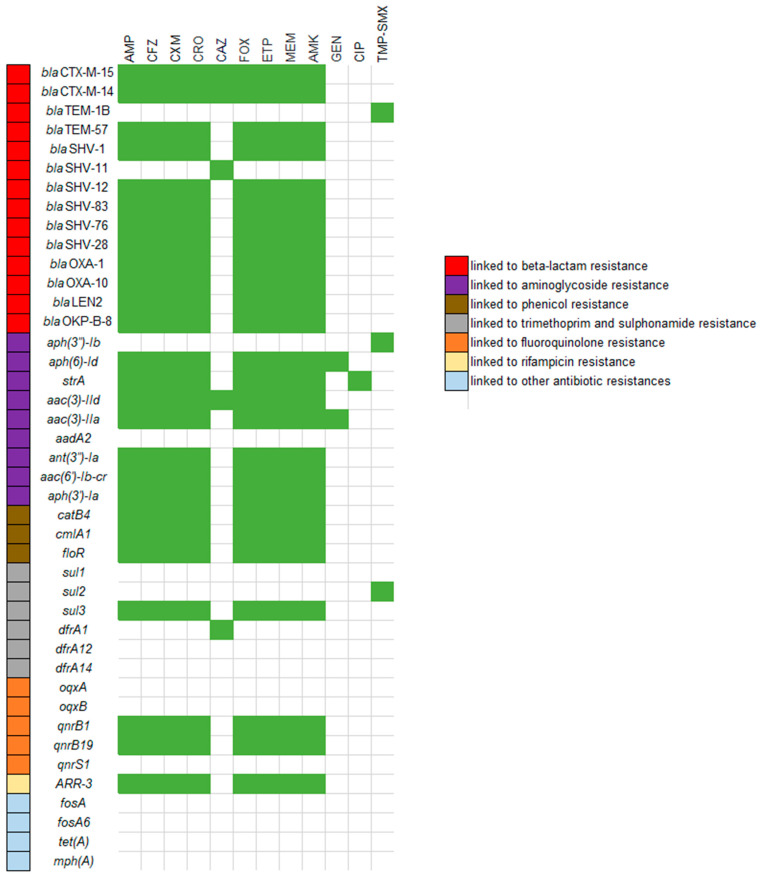
Association matrix between genotype and resistance phenotype. Green squares represent statistically significant associations. Ampicillin, AMP; cefazolin, CFZ; cefuroxime, CXM; ceftriaxone, CRO; ceftazidime, CAZ; cefoxitin, FOX; ertapenem, ETP; meropenem, MEM; amikacin, AMK; gentamicin, GEN; ciprofloxacin, CIP; trimethoprim/sulfamethoxazole, TMP-SMX.

Of the 14 genes conferring resistance to beta-lactams, 10 genes were associated with phenotypic resistance to all the beta-lactams tested, excluding ceftazidime, and to amikacin. The *blaS*HV-11 gene was only associated with phenotypic resistance to ceftazidime. The two *bla*CTX-M-types were associated with phenotypic resistance to all the beta-lactams tested and to amikacin. Of the nine genes linked to aminoglycoside resistance, seven were associated with phenotypic resistance to amikacin, of which two genes were also associated with phenotypic gentamycin resistance. An association was found between resistance to trimethoprim/sulfamethoxazole and the presence of *sul2* (Figure 3).

**Figure 4:**
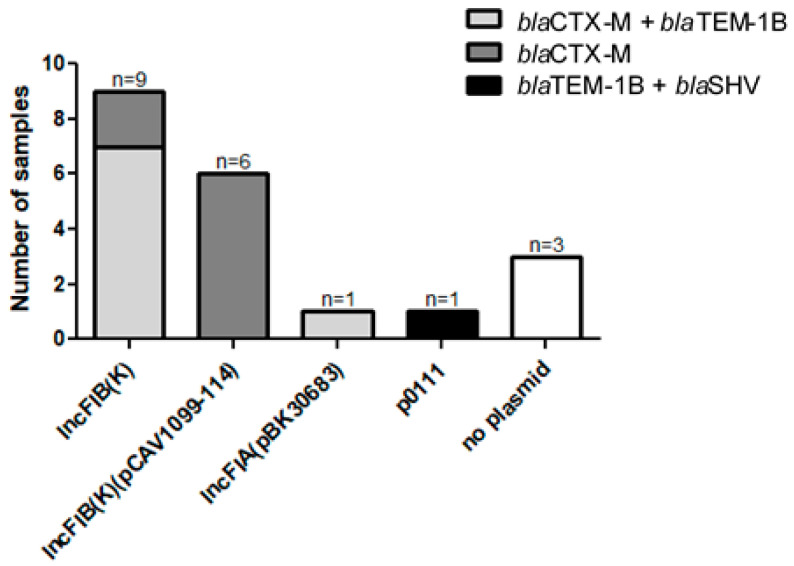
Distribution of ESBL genes per plasmid types among the ESBL-producing isolates.

**Figure 5:**
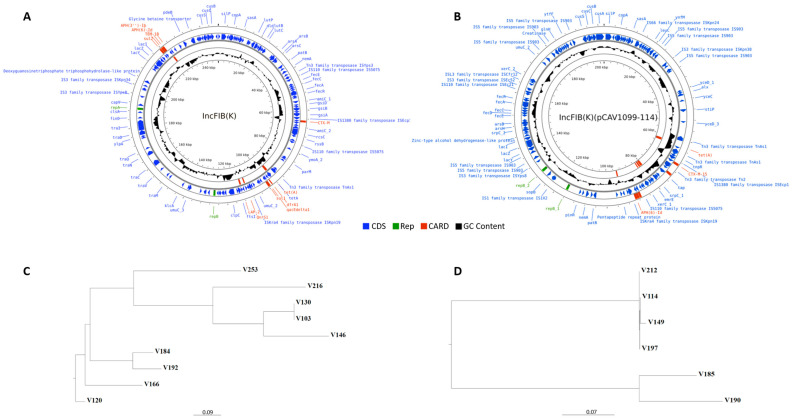
Plasmid types and rooted distance trees of ESBL-positive isolates. (**A**,**B**) Backbone structure of a representative plasmid sequence of (**A**) the IncFIB(K) plasmid type and (**B**) the IncFIB(K)(pCAV1099-114) plasmid type. On plasmid type IncFIB(K), the CTX-M gene refers to either CTX-M-14 or CTX-M-15, depending on the isolate. Blue, CDS; green, rep; red, CARD (Comprehensive Antibiotic Resistance Database); black, GC content. (**C**,**D**) Rooted distance tree of ESBL-positive isolates carrying the (**C**) IncFIB(K) plasmid type and (**D**) IncFIB(K)(pCAV1099-114) plasmid type.

The distribution of ESBL genes carried on each plasmid type among all ESBL-producing isolates is presented in Figure 4. All isolates, excluding three, carried plasmids containing ESBL genes; the gene most frequently carried by plasmids was *bla*CTX-M, followed by *bla*TEM. The IncFIB plasmid replicon group was identified in 75% of the ESBL-producing isolates. Two plasmid types, IncFIB(K) and IncFIB(K)(pCAV1099-114), were most common in the ESBL sample. Plasmid type IncFIB(K) appeared in nine isolates (45%), of which seven isolates carried the combination of *bla*CTX-M+*bla*TEM-1B genes on the plasmid and two carried only *bla*CTX-M-15. Plasmid type IncFIB(K)(pCAV1099-114) was identified in six isolates (30%) and harbored *bla*CTX-M. An overview of the plasmid types most frequently found, IncFIB(K) and IncFIB(K)(pCAV1099-114), is displayed in Figure 5A and Figure 5B, respectively. IncFIB(K) was associated with a variety of resistance gene cassette arrays among the nine isolates carrying IncFIB(K) (Figure 5C), whereas IncFIB(K)(pCAV1099-114) was more homogeneous among the six isolates carrying the plasmid (Figure 5D).

**Figure 6:**
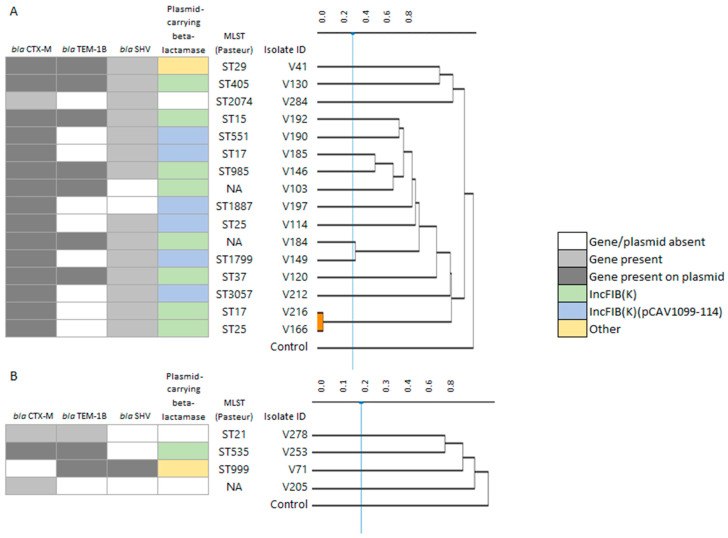
Genotypic and phenotypic characteristics of ESBL-producing isolates. (**A**) *K. pneumoniae* isolates and (**B**) *E. coli* isolates. The dendrogram on the right displays the relative distance of the isolates according to Fourier-transform infrared (FT-IR) typing.

The molecular epidemiology of the ESBL isolates was very heterogeneous. Of the 16 ESBL-producing *K. pneumoniae* isolates, multilocus sequence typing (MLST) identified 12 distinct sequence types (ST). One *K. pneumoniae* isolate belonged to an emerging epidemic clone ST named ST15, two belonged to ST17, and two isolates belonged to ST25. The remaining *K. pneumonia* isolates each belonged to a different ST (ST2074, ST37, ST1887, ST29, ST405, ST985, ST1799, ST551, ST3057, and two undetermined STs). Fourier-transform infrared (FT-IR) spectroscopy typing of *K. pneumoniae* did not reveal any clusters, except for one cluster comprising two ESBL-producing *K. pneumoniae* isolates with a similar genetic and resistance profile but different STs (ST17 and ST25), both isolated from packaged sprouts collected on different days from different market types (Figure 6A). Likewise, MLST revealed high diversity among the four ESBL-producing *E. coli* isolates (ST535, ST999, ST21, and one undetermined ST). The FT-IR typing of *E. coli* did not yield any clusters, confirming the diversity among the *E. coli* strains (Figure 6B).

## 3. Discussion

The vegetable industry in Israel includes a wide variety of crops, with a production volume of 1.2 million metric tons in 2021 [22]. While studies of ESBL/AmpC-producing bacteria present in vegetables have been conducted in Europe, Africa, Asia and America [23], this is the first such study conducted in Israel.

Currently, nearly 90% of Israel’s treated wastewater is reused for irrigation purposes [24]. Furthermore, Israel has a high rate of 3GCR (>20%) in community settings and ESBL carriers in the community are common [13]. Nevertheless, the prevalence of ESBL in vegetables in Israel obtained from our study is low. Other countries have also demonstrated relatively high 3GCR rates in community settings but a relatively low prevalence of ESBL/AmpC in vegetables. For example, countries with high 3GCR, such as South Korea, Romania, Brazil, and Nepal [25], exhibited low (<10%) [20,26] or medium (10–20%) [14,27] ESBL/AmpC prevalence in vegetables. Similarly, countries with a medium 3GCR rate, such as the United States and United Kingdom [25], have low ESBL/AmpC prevalence in vegetables [15,28]. In contrast, a correlation between 3GCR prevalence in human isolates and prevalence of ESBL/AmpC in vegetable samples was found in countries such as the Netherlands (low 3GCR [25] and low ESBL/AmpC prevalence [16,17,29]), India (high 3GCR [25] and high ESBL prevalence (>20%) [18]), and Spain (medium 3GCR [25] and medium ESBL prevalence [19]).

The ESBL prevalence in our study was 6.6%, whereas other studies reported a wide range of prevalence between 0.06 and 25.4% [14,15,16,17,18,19]. The ESBL-positive *E. coli* strains comprised 1.3% of our sample, while previous studies found an ESBL *E. coli* prevalence of 0–13.8% [26,27,28]. The percentage of AmpC-producing isolates in our study was 20.9%, and in contrast, previous studies reported a range of 0.47–14.3% [15,17,20,21]. We demonstrated that the prevalence of ESBL/AmpC-producing isolates was associated with the types of vegetables, packaging, and markets; therefore, differences in results between these various studies are most likely explained by these factors. This supports the proposition by van Hoek et al. that the wide variety in prevalence of ESBL-producing isolates reported in different studies was due to differences in vegetable types [17]. Moreover, sample size, testing method, hygiene awareness in each country, and local beta-lactamase prevalence may impact the results as well.

*K. pneumoniae* was the predominant ESBL-producing bacterium in our study, followed by *E. coli*. This observation may suggest that ESBL-producing *Klebsiella* spp. are more capable of spreading and persisting in the environment than ESBL-producing *E coli*, as proposed previously [30]. Indeed, the prevalence of ESBL-producing *Klebsiella* spp. in community-acquired infections has been shown to be steadily increasing [31].

ESBL was scarcely found in climbing vegetable plants, likely because they grow above ground and therefore are less exposed to soil and contaminated fertilizer or wastewater, thus reducing the possibility of contamination [12]. This finding supports previous work that revealed negligible microbial risk in cucumbers and tomatoes [32]. In our study, the majority of AmpC isolates came from leafy green vegetables, in agreement with the World Health Organization’s statement that leafy vegetables were the greatest concern in terms of microbiological hazards in vegetables [33].

Most of the ESBL-producing bacteria in our study exhibited resistance to fluoroquinolones. The proportion of ESBL-producing isolates’ resistance to fluoroquinolones has increased over time [34]. In studies conducted 20 years ago, 45% of ESBL-producing isolates were resistant to fluoroquinolones [35], compared to 70% in our study. Worldwide, the majority of ESBL-producing isolates are susceptible to carbapenems; thus, carbapenems are the mainstay of treatment against infections caused by ESBL-producing pathogens [36]. All ESBL-producing isolates in our study were susceptible to carbapenems. They were also all susceptible to amikacin, as previously reported among hospitalized and community patients [37].

In our study, all but one *E. coli* ESBL-positive isolate produced CTX-M. Previous studies have also found that CTX-M was the most predominant type of ESBL among bacteria found in vegetables [18,23,29]. CTX-M-15 was the prevalent allele, constituting 80% of our sample, consistent with previous studies [36,38].

The dissemination of ESBL genes can be attributed to highly transmissible plasmids. In our ESBL sample, 85% of the isolates originating from fresh vegetables carried a plasmid coding for beta-lactam resistance, indicating a threat of resistance dissemination by horizontal gene transmission via plasmids. Among our ESBL-producing isolates, the IncFIB plasmid was the most predominant replicon group, in agreement with previous reports on vegetable samples [14,39]. Moreover, the IncFIB plasmid group is prevalent in aquatic environments [40] and in clinical settings [41], indicating the vast transmission and dissemination of the IncFIB plasmid. The high prevalence of the IncF plasmid may be attributed to the presence of addiction systems, i.e., systems which increase plasmid stability by ensuring that only cells containing the plasmid survive, whereas those that have lost the plasmid are eliminated. Therefore, the IncF plasmid serves as an efficient genetic platform to spread ESBL resistance [8].

While in clinical settings it is more common for isolates to be clonally related [36], in the community, isolates are varied, as shown by the wide genetic diversity among the ESBL-producing isolates in our study. The broad assortment of resistance genes found in all the ESBL-positive isolates, specifically resistance to clinically relevant antimicrobials, is a concerning finding. One ESBL-producing *K. pneumoniae* belonged to a high-risk epidemic clone associated with nosocomial infections in humans (ST15) [42], suggesting dispersal of ESBL genes via raw vegetables.

Beta-lactam resistance poses a challenge for therapeutic treatment, and results in prolonged hospital stays and higher mortality rates [43]. Food screening for ESBL/AmpC-producing bacteria is essential to protect health. Consuming raw contaminated vegetables may result in the transfer of resistance genes located on mobile elements, such as ESBL genes, to opportunistic intestinal pathogens [44,45,46]. Furthermore, extensive exposure to AmpC-producing bacteria may result in colonization [1]. The US Food and Drug Administration has issued a guidance document for the produce industry that outlines principles and practices to minimize microbial food safety hazards during fresh fruit and vegetable production, but the guidance is a mere recommendation and not a regulation that can be enforced [47].

Our study has several limitations. First, while vegetables from a variety of markets were investigated, we did not include vegetables collected directly from farms or from produce factories. Future studies should consider testing those sites in order to provide information on critical points in the farm-to-consumer continuum. Second, we tested for the presence of ESBL/AmpC in fresh vegetables, but did not quantify the bacterial load (CFU) found in the vegetable samples. Third, it was beyond the scope of this study to examine the extent of the public health risks from consumption of ESBL/AmpC via fresh vegetables.

## 4. Materials and Methods

### 4.1. Study Design and Sample

In July and August 2021, 301 vegetable samples were purchased from 70 unique markets in the central region of Israel, all within a 60 km radius. The markets included supermarket chains, i.e., large grocery stores which offer a wide variety of products (*n* = 17); local minimarkets, i.e., small grocery stores which sell a limited selection of basic food products (*n* = 20); and outdoor markets, i.e., open-air markets with individual stands (*n* = 33 different stands from three outdoor markets). From each market, we collected different types of vegetables, based on availability. The vegetable types included climbing vegetables, i.e., vegetables grown vertically by supports, such as trellises (tomatoes and cucumbers); leafy greens, i.e., plant leaves eaten as food (parsley, cilantro, and lettuce); and vegetables grown in environment-controlled beds, i.e., vegetables grown on a prepared bed with controlled temperature, soil mixture type, and light conditions (sprouts and mushrooms). Of the sample, 132 vegetables were packaged in sealed plastic bags and 169 were unpackaged.

### 4.2. Laboratory Methods

#### 4.2.1. Specimen Processing

After purchase, the samples were transported to the laboratory in coolers. All samples were immediately stored in plastic bags at 4 °C and processed within three hours of their acquisition. A total of 25–40 g of each unwashed vegetable sample from a single market was sliced and inoculated in 30 mL Brain Heart Infusion broth (Hylabs, Rehovot, Israel). Following overnight incubation under aerobic conditions at 35 ± 2 °C, the broth was subcultured on a selective screening agar plate, CHROMagar ESBL (Hylabs, Rehovot, Israel). Identification of the species level and antibiotic susceptibilities were determined with VITEK^®^ MS or VITEK^®^ 2, using the GN card for identification and the AST-N395 card for susceptibilities (bioMe’rieux SA, Marcy l’Etoile, France). The antibiotics tested were ampicillin, cefazolin, cefuroxime, ceftriaxone, ceftazidime, cefoxitin, ertapenem, meropenem, amikacin, gentamicin, ciprofloxacin, and trimethoprim/sulfamethoxazole.

#### 4.2.2. Disk Diffusion

Phenotypic ESBL production was confirmed using the disk diffusion method with clavulanic acid (Oxoid Ltd., Hants, UK), according to CLSI M100 2023 [48]. The antibiotics tested were ceftazidime (30 µg, CAZ), ceftazidime-clavulanate (30/10 µg, CAZ-CLA), cefotaxime (30 µg, CTX), and cefotaxime-clavulanate (30/10 µg, CTX-CLA). Interpretation of the results was undertaken according to the CLSI M100 guidelines. Quality control strains were tested as required by CLSI [48]. Isolates negative for ESBL were further tested for the presence of AmpC using the MASTDISCS^®^ *Combi* AmpC and ES*β*L Detection Set (Mast Group Ltd., Merseyside, United Kingdom) and the results were interpreted according to the manufacturer’s instructions.

#### 4.2.3. FT-IR Typing

FT-IR is a typing method which generates a spectrum based on the absorption of infrared light of the bacterial composition. These spectra reflect the specific signatures of specimens and can be grouped into a cluster according to spectra similarity [49]. FT-IR was performed as described previously [49]. Briefly, isolates were grown at 35 ± 2 °C on blood agar plates (HyLabs, Rehovot, Israel) and samples were prepared in quadruplicate according to the IR Biotyper (Bruker, Leipzig, Germany) manufacturer’s instructions. The spectra and cluster analysis were generated by the OPUS software (Bruker, version 7.5). The cut-off ranges recommended by the manufacturer for *E. coli* and *K. pneumoniae* were 0.15-0.18 and 0.2–0.25, respectively. The ESBL-producing isolates were subjected to FT-IR typing with the addition of two unrelated known ESBL-producing *E. coli* and *K. pneumoniae* from our collection, serving as controls.

#### 4.2.4. WGS and Bioinformatics Analysis

The ESBL-positive isolates were subjected to WGS. DNA extraction was performed on the MICROLAB Nimbus workstation (Hamilton, Reno, NV, USA) with a STARMag 96 Universal Kit (Seegene, Seoul, Republic of Korea). Samples were sequenced with Oxford Nanopore at SNPsaurus, Colorado, Oregon. Resistance genes were detected using ResFinder (https://cge.cbs.dtu.dk/services/ResFinder/—version 25.4.23, accessed on 7 August 2023). Pangenome analysis of all genomes was performed with version 3.12.0 of Roary. The resulting core genome alignment was then used to generate a phylogenetic maximum likelihood tree using version 8.2.12 of RAxML with the GTRGAMMA model. Genes were classified as plasmid-related if they were located on the same contig as the plasmid *rep* gene, whereas genes that were not located on the plasmid contig were classified as neither plasmid nor chromosomal. A polymerase chain reaction (PCR) of *bla*CTX-M, *bla*TEM, and *bla*SHV was performed for two ESBL-negative isolates serving as negative controls (an AmpC-positive isolate and an ESBL/AmpC-negative *Acinetobacter* spp. isolate), using specific primers [50,51,52] (Table 2).

#### 4.2.5. Statistical Analysis

Odds ratios and their 95% confidence intervals were calculated to compare the prevalence of AmpC- or ESBL-producing bacteria between vegetable types, packaging, and market types. The association between phenotype and genotype was assessed using a chi-square test. All the analyses were performed in Stata version 14.2 (StataCorp, College Station, TX, USA).

## 5. Conclusions

In our study, we detected ESBL/AmpC-producing bacteria in fresh vegetables. Further analysis revealed that most of the ESBL genes were carried on plasmids. The presence of ESBL/AmpC resistance in bacteria from vegetables highlights the need for food screening and monitoring of antimicrobial resistance in fresh produce, including plasmid identification, to increase food safety and limit the dispersal of resistance genes. The plasmid-borne ESBL and AmpC in bacterial populations in fresh vegetables constitute a reservoir of antibiotic resistance genes and may contribute to global resistance dissemination in the community. Further studies are needed to evaluate the impact of the ESBL/AmpC-producing bacteria found in raw vegetables on human health.

## Figures and Tables

**Table 1:** Prevalence of AmpC- or ESBL-producing *Enterobacterales* in vegetables.

	AmpC-Positive		ESBL-Positive	
	N (%)	Odds Ratio (95% CI)	N (%)	Odds Ratio (95% CI)
Vegetable type				
Climbing vegetables	18/123 (14.6%)	Reference	3/123 (2.4%)	Reference
Leafy greens	40/121 (33.1%)	2.88 (1.54–5.39)	12/121 (9.9%)	4.40 (1.21–16.02)
Environment-controlled beds	5/57 (8.8%)	0.56 (0.20–1.59)	5/57 (8.8%)	3.85 (0.89–16.69)
Packaging				
Unpackaged	34/169 (20.1%)	Reference	7/169 (4.1%)	Reference
Packaged	29/132 (22.0%)	1.12 (0.64–1.95)	13/132 (9.8%)	2.53 (0.98–6.53)
Market type				
Outdoor market	21/89 (23.6%)	Reference	10/89 (11.2%)	Reference
Supermarket	17/114 (14.9%)	0.57 (0.28–1.16)	8/114 (7.0%)	0.60 (0.23–1.58)
Minimarket	25/98 (25.5%)	1.11 (0.57–2.16)	2/98 (2.0%)	0.16 (0.04–0.77)

**Table 2:** List of primers used in this study.

Gene	Forward Primer	Reverse Primer	Reference
*bla*CTX-M	RGMAGYGYRMCGCTKYATGCSC	ARTARGTSACCAGAAYVAGCGG	[51]
*bla*TEM	TCAACATTTCCGTGTCG	CTGACAGTTACCAATGCTTA	[50]
*bla*SHV	ATTTGTCGCTTCTTTACTCGCC	TTCACCACCATCATTACCGACC	[52]

## Data Availability

The datasets used and/or analyzed during the current study are available from the corresponding author on reasonable request.

## Supplementary Materials

The following supporting information can be downloaded at: https://www.mdpi.com/article/10.3390/antibiotics12101528/s1, Table S1: Phenotypic resistance profile of EBSL-producing isolates; Table S2: Resistance gene profile of EBSL-producing isolates.
