# Supplementary material for: Occurrence, Typing, and Resistance Genes of ESBL/AmpC-Producing Enterobacterales in Fresh Vegetables Purchased in Central Israel
Source: Antibiotics (Basel). 2023 Oct 11;12(10):1528. doi: 10.3390/antibiotics12101528 (PMC10604292; doi:10.3390/antibiotics12101528)
Supplement: Supplementary file 1 [file antibiotics-12-01528-s001.zip › antibiotics-2618739-supplementary.pdf]

Supplementary

Table S1: Phenotypic resistance profile of EBSL-producing isolates

Antibiotic class	Beta-lactam								Aminoglycoside		Fluoroquinolone	
Isolate ID	AMP	CFZ	CXM	CRO	CAZ	FOX	ETP	MEM	AMK	GEN	CIP	TMP-SMX
V41	R	R	R	R	R	S	S	S	S	S	R	R
V130	R	R	R	R	I	S	S	S	S	S	R	R
V284	R	R	R	R	R	S	S	S	S	S	S	S
V192	R	R	R	R	R	S	S	S	S	R	R	R
V190	R	R	R	R	R	S	S	S	S	S	R	R
V185	R	R	R	R	R	S	S	S	S	S	R	S
V146	R	R	R	R	R	S	S	S	S	R	R	R
V103	R	R	R	R	R	S	S	S	S	S	R	R
V197	R	R	R	R	I	S	S	S	S	S	I	R
V114	R	R	R	R	R	S	S	S	S	S	R	R
V184	R	R	R	R	I	S	S	S	S	R	R	R
V149	R	R	R	R	R	S	S	S	S	S	R	R
V120	R	R	R	R	S	S	S	S	S	S	I	R

V212	R	R	R	R	I	S	S	S	S	S	I	R
V216	R	R	R	R	S	S	S	S	S	R	R	S
V166	R	R	R	R	S	S	S	S	S	R	R	S
V278	R	R	R	R	I	S	S	S	S	S	I	R
V253	R	R	R	R	S	S	S	S	S	S	R	R
V71	R	R	R	R	I	S	S	S	S	R	R	R
V205	R	R	R	R	R	S	S	S	S	S	I	S

AMP, ampicillin; CFZ, cefazolin; CXM, cefuroxime; CRO, ceftriaxone; CAZ, ceftazidime; FOX, cefoxitin; ETP, ertapenem; MEM, meropenem; AMK, amikacin; GEN, gentamicin; CIP, ciprofloxacin; TMP-SMX, trimethoprim/Sulfamethoxazole. Susceptibilities were interpreted according to CLSI M100-S27

Table S2: Resistance gene profile of EBSL-producing isolates

Isolate ID	Species	Beta-lactam	Aminoglycoside	Fluoroquinolone	Fosfomycin	Sulfonamide	Trimethoprim	Tetracycline	Macrolide	Phenicol	Quaternary ammonium
V41	*K. pneumoniae*	*bla*CTX-M-15, *bla*TEM-1B, *bla*SHV-187	*aph(3'')-Ib, aph(6)-Id*	*OqxA, OqxB, qnrB1*	*fosA*	*sul2*	*dfrA14*	*tet(A)*			
V130	*K. pneumoniae*	*bla*CTX-M-15, *bla*TEM-1B, *bla*SHV-76	*aac(6')-Ib-cr, aph(3'')-Ib, aph(6)-Id*	*OqxA, OqxB, qnrB1, aac(6')-Ib-cr*	*fosA*	*sul2*	*dfrA14*	*tet(A)*		*catB3*	
V284	*K. pneumoniae*	*bla*CTX-M-15, *bla*SHV-11*	*aph(3'')-Ib, aph(6)-Id*	*OqxA, OqxB*	*fosA*						
V192	*K. pneumoniae*	*bla*CTX-M-15, *bla*TEM-1B, *bla*SHV-28	*aadA2, aac(3)-IIa*	*OqxA, OqxB*	*fosA*	*sul1*	*dfrA12*		*mph(A)*	*floR*	*qacE*
V190	*K. pneumoniae*	*bla*CTX-M-15, *bla*SHV-172	*aph(6)-Id, aadA2, aph(3')-Ia, aph(3'')-Ib*	*OqxA, OqxB, qnrS1*	*fosA*	*sul1, sul2*	*dfrA12*		*mph(A)*		*qacE*
V185	*K. pneumoniae*	*bla*CTX-M-15, *bla*SHV-11*	*aph(3'')-Ib, aph(6)-Id*	*OqxA, OqxB, qnrS1*	*fosA*	*sul2*		*tet(A)*			
V146	*K. pneumoniae*	*bla*CTX-M-15, *bla*TEM-1B, *bla*SHV-187	*aac(3)-IIa, aac(6')-Ib-cr, aph(3'')-Ib, aph(6)-Id*	*OqxA, OqxB, qnrB19, aac(6')-Ib-cr*	*fosA*	*sul2*	*dfrA14*	*tet(A)*		*catB3*	

V103	*K. pneumoniae*	*bla*CTX-M-15, *bla*TEM-1B	*aph(3'')-Ib, aph(6)-Id*	*OqxA, OqxB, qnrB1*	*fosA*	*sul2*	*dfrA14*	*tet(A)*			
V197	*K. pneumoniae*	*bla*CTX-M-15	*aadA2, aph(3')-Ia, aph(3'')-Ib, aph(6)-Id*	*OqxA, OqxB, qnrS1*	*fosA*	*sul1, sul2*	*dfrA12*		*mph(A)*		*qacE*
V114	*K. pneumoniae*	*bla*CTX-M-15, *bla*SHV-11*	*aadA2, aph(3')-Ia, aph(3'')-Ib, aph(6)-Id*	*OqxA, OqxB, qnrS1*	*fosA*	*sul1, sul2*	*dfrA12*		*mph(A)*		*qacE*
V184	*K. pneumoniae*	*bla*CTX-M-15, *bla*TEM-1B, *bla*SHV-1	*aac(3)-IIa, aac(6')-Ib-cr, aph(3'')-Ib*	*OqxA, OqxB, qnrB1, aac(6')-Ib-cr*	*fosA*	*sul2*	*dfrA14*	*tet(A)*		*catB3*	
V149	*K. pneumoniae*	*bla*CTX-M-15, *bla*SHV-28	*aadA2, aph(3')-Ia, aph(3'')-Ib, aph(6)-Id*	*OqxA, OqxB, qnrS1*	*fosA*	*sul1, sul2*	*dfrA12*		*mph(A)*		*qacE*
V120	*K. pneumoniae*	*bla*CTX-M-14, *bla*TEM-1B, *bla*SHV-11*	*aph(3'')-Ib, aph(6)-Id*	*OqxA, OqxB, qnrS1*	*fosA*	*sul1, sul2*	*dfrA1*	*tet(A)*			*qacE*
V212	*K. pneumoniae*	*bla*CTX-M-15, *bla*SHV-33	*aadA2, aph(3'')-Ib, aph(6)-Id*	*OqxA, OqxB, qnrS1*	*fosA*	*sul1, sul2*	*dfrA12*	*tet(A)*	*mph(A)*		*qacE*
V216	*K. pneumoniae*	*bla*CTX-M-14, *bla*SHV-11*	*aac(3)-IId*	*OqxA, OqxB, qnrS1*	*fosA*	*sul1*	*dfrA1*	*tet(A)*			*qacE*
V166	*K. pneumoniae*	*bla*CTX-M-14, *bla*SHV-11*	*aac(3)-IId*	*OqxA, OqxB, qnrS1*	*fosA*	*sul1*	*dfrA1*	*tet(A)*			*qacE*

V278	*E. coli*	*bla*CTX-M-15, *bla*TEM-1B	*aph(6)-Id*, *aph(3'')-Ib*	*qnrS1*		*sul2*	*dfrA14*	*tet(A)*			
V253	*E. coli*	*bla*CTX-M-15, *bla*TEM-1B	*aph(3'')-Ib*, *aph(6)-Id*	*qnrS1*		*sul2*	*dfrA14*	*tet(A)*			
V71	*E. coli*	*bla*TEM-1B, *bla*SHV-12	*aac(3)-IIa*, *aadA1*, *aadA2*	*qnrS1*		*sul1*, *sul3*	*dfrA12*	*tet(A)*	*mph(A)*	*floR*	*qacE*, *qacL*
V205	*E. coli*	*bla*CTX-M-15		*qnrS1*							
